# The Meaning of "Infectious"

**Published:** 1916-03-18

**Authors:** 


### THE MEANING OF "INFECTIOUS."

The inquiry here briefly to be discussed is not a philological or historical or doctrinal one. These aspects of the subject have their interest and value, but they find their opportunity elsewhere. What we wish to ask is—What interpretation in the world of practical affairs is to be placed on the word "infectious" as this is applied to a particular disease. Parliament has a short way with the difficulty, as is its privilege. All that is necessary for legislative purposes is to set out a list of diseases, to read this three times in one House and three times in the other, and forthwith the matter is settled. Whatever is found within this list is henceforth regarded by the legal eye as "infectious," while all others escape the ban. Mr. Bumble had a strong opinion of the "law," and this we do not propose either to endorse or to condemn. All we wish to add is that, while Parliamentary definitions may be as convenient as they are powerful in the sphere to which they apply, they by no means settle controversy in other areas. Thus in the matter now before us, though in a particular Act of Parliament a number of diseases are duly scheduled as "infectious," it is certain that the problems these present in relation both to the individual sufferer and to the public health are far from being concluded. Municipal and other official persons do not always realise this. Thus but a few years ago a public personage addressing his peers "somewhere" in the neighbourhood of Spring Gardens claimed that the problem of tuberculosis was a matter which could present no difficulty. The disease had recently been proclaimed as "infectious" by the Local Government Board, and the same authority applied the same term to scarlet fever. But health committees had long been accustomed to deal successfully with scarlet fever, and all they had now to do was to apply exactly the same methods to tuberculosis. A truly ingenuous argument, strongly based on a legal definition, but long since subjected to ridicule in the bitter school of experience!

If the Parliamentary definition is not an adequate guide, neither, must it be said, is the extreme scientific one, at least when it is appealed to by minds undisciplined by scientific training. From some lay discussions it would be concluded that the mere fact that a given disease arises from the presence of a micro-organism is sufficient to warrant the application to the sufferers of isolation and all the other rigours of preventive medicine. Public dealing with tuberculosis has done something to correct this error, but has not fully destroyed it. And the argument still occasionally takes a prominent flight, more especially when cases of some individual disease are unusually numerous or when an outbreak assumes a quasi-epidemic character. Rigorously applied, this claim would mean that quite a number of diseases now dealt with by ordinary measures ought in every instance to be placed under the ban of "infectious," and should be subjected to the conditions which this term implies. The brief fact is that at present we must order our conduct towards diseases by reference neither to Parliamentary wisdom nor to bacteriological science, but strictly according to the teachings of experience. If in practice a disease does not as a fact spread from person to person, it is uneconomical and unnecessary to apply to it conditions which both penalise the patient and involve expense, and this whether it is or is not dignified in a Parliamentary schedule and whether it is or is not of microbic causation.

